# Temporary Gasserian ganglion stimulation utilizing SNM electrode in subacute herpetic trigeminal neuralgia

**DOI:** 10.3389/fneur.2024.1435272

**Published:** 2024-07-17

**Authors:** Jiejie Niu, Chenhui Wang, Xing Wang, Guijun Lu

**Affiliations:** ^1^Department of Pain Management, Beijing Tsinghua Changgung Hospital, School of Clinical Medicine, Tsinghua University, Beijing, China; ^2^Department of Anesthesiology, Sanbo Brain Hospital, Capital Medical University, Beijing, China; ^3^Department of Radiotherapy, Beijing Tsinghua Changgung Hospital, School of Clinical Medicine, Tsinghua University, Beijing, China

**Keywords:** Gasserian ganglion stimulation, herpetic trigeminal neuralgia, electrode, neuropathic pain, neuromodulation

## Abstract

**Objective:**

Gasserian ganglion stimulation (GGS) is a neuromodulation technique that has been extensively applied in treating postherpetic trigeminal neuralgia. However, permanent implantation of GGS was preferred in most treatment approaches. Few studies have investigated temporary GGS for the treatment of acute/subacute herpetic trigeminal neuralgia. Moreover, previous research has reported lead dislocation when utilizing traditional electrodes, which was associated with poor pain relief. In GGS research, preventing the accidental displacement of lead following implantation has consistently been a primary objective.

**Methods:**

We report a case of a 70-year-old woman with subacute herpetic trigeminal neuralgia who underwent temporary GGS for 14 days utilizing a sacral neuromodulation (SNM) quadripolar-tined lead. Computed tomography-guided percutaneous foramen ovale (FO) puncture and temporary SNM electrode implantation were performed during the surgery. A telephone interview was conducted to record a 12-month follow-up.

**Results:**

At admission, zoster-related trigeminal pain severity was assessed to be 9/10 on the visual analog scale (VAS). After a 14-day GGS treatment, the pain assessed on the VAS score reduced to 1/10 at discharge but increased to 4/10 at the 12-month follow-up after surgery. Additionally, the anxiety level improved from 58 points to 35 points on the Self-Rating Anxiety Scale (SAS), and the depression level improved from 62 points to 40 points on the Self-Rating Depression Scale (SDS). The Physical Component Summary score of the 12-item Short-Form Health Survey (SF-12) increased from 33.9 to 47.0, and the Mental Component Summary (MCS) score of the SF-12 increased from 27.4 to 41.9.

**Conclusion:**

Temporary GGS might be a potentially effective treatment for subacute herpetic trigeminal neuralgia, and an SNM electrode might be a good choice for reducing the risk of dislocation.

## 1 Introduction

Herpetic neuralgia is a typical form of neuropathic pain characterized by intense burning, sharp or lightning-like sensation, and/or hyperalgesia along the involved nerve distribution (1). The annual morbidity rate of herpes zoster has been estimated to be 3.9 to 42 per 100,000 person-years, and the dermatome of herpes zoster varies in different patients (2), which can be commonly observed in the distribution of the unilateral thoracic area, the lumbosacral area, and the neck area. Herpes zoster is a disease that mainly affects the elderly (3), with 9–34% of cases eventually converting to postherpetic neuralgia (PHN) (4). The initial treatment for herpetic neuralgia is generally based on medications, including antiviral drugs, anticonvulsants, and antidepressants. Nonetheless, many patients are left with unrelieved severe neuropathic pain and do not respond to medication therapy (5). Herpetic neuralgia involving the trigeminal nerve is also a type of herpes zoster-related pain in the orofacial region, which accounts for approximately 15–20% of cases (4). Gasserian ganglion is the largest sensory ganglion of the fifth cranial nerve and contains three major divisions of the trigeminal nerve: the ophthalmic nerve (V1), the maxillary nerve (V2), and the mandibular nerve (V3). It plays an important role in processing sensory information, including pain sensory information, and peripheral and central sensitization (6). When the dormant and latent herpes zoster virus invades the Gasserian ganglion, the virus reactivates and replicates as the immune system becomes immunocompromised. This phenomenon is associated with peripheral nerve lesions and an infected ganglion, manifesting as neuronal edema and necrosis (7, 8). These severe inflammatory reactions in the Gasserian ganglion eventually result in neuropathic pain. Traditional spinal cord stimulation (SCS), a neuromodulation technique that has been widely applied in the treatment of refractory neuropathic pain with obvious analgesic effects (9, 10), has little therapeutic effect in herpetic trigeminal neuralgia patients. Although some studies reported that epidural stimulation at the C1–C2 level could generate facial paresthesia (11–13), inadequate coverage of the pain region would occur inevitably if herpes zoster invaded the V1 or V2 branch (4, 14). Peripheral nerve stimulation (PNS) is also an effective treatment for herpetic trigeminal neuralgia (15–17), which provided at least 50% pain relief in 70–80% of patients, but these studies only included patients with mono branch lesions. It is difficult for patients with multiple branch lesions of the trigeminal nerve to benefit from one-lead PNS. Gasserian ganglion stimulation (GGS) was first demonstrated in 1980 with a significant outcome, and a bipolar plate was implanted by employing the open craniotomy procedure (18). Later, percutaneous GGS with electrodes of various shapes and sizes was gradually developed, which has been a promising treatment in refractory trigeminal neuropathy. Nevertheless, there are still some issues that require further investigation. First, the bipolar plate or 8-contact lead was being extensively applied in the treatment of trigeminal neuropathy at the level of Gasserian ganglion neuromodulation, but a high incidence of electrode dislocation and dysesthesia was reported. To solve the problem, Medtronic developed a custom-made tripolar-tined lead (19, 20), but it was not available in China, which limited the use of this electrode. Second, if the trial stimulation was satisfactory, permanent electrode implantation was worth considering to ensure good pain control; however, this put a great financial burden on patients. It is necessary to figure out whether short-term GGS can achieve the same level of relatively long-term pain relief upon prompt stimulation. Third, most of the studies focused on the treatment of permanent GGS for stroke- or surgery-induced trigeminal neuropathy, and to date, few studies have investigated the efficacy of temporary GGS for acute herpetic neuralgia (AHN) or subacute herpetic neuralgia (SHN).

**Figure 1:**
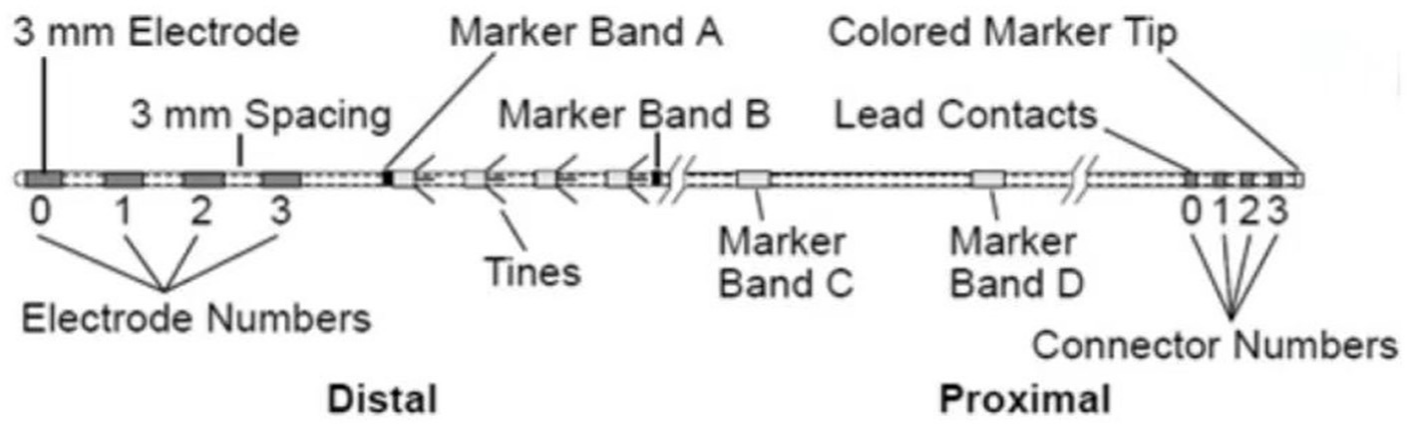
A quadripolar-tined SNM lead, Model 3889, Medtronic.

The electrode for sacral neuromodulation (SNM) is a quadripolar-tined lead (Model 3889 lead, Medtronic interStim System) first designed to be implanted adjacent to the sacral nerve (21). This has become one of the most accepted leads for SNM treatment in the field of functional urology. The Model 3889 lead (Figure 1) contains four cylindrical electrodes of equal length and tines that are designed to prevent electrode dislocation after implantation. However, it has not been applied in the field of temporary GGS for the treatment of herpetic trigeminal neuralgia.

Therefore, we report a case of a 70-year-old woman with SHN involving the left V2 and V3 branches of the trigeminal nerve who successfully underwent temporary GGS using an SNM electrode.

## 2 Case description

A 70-year-old woman presented to our pain clinic with left facial pain that began ~2 months earlier. The patient complained of burning, spontaneous paroxysmal pain with sensitivity to touch in the regions of the left zygomatic zone and the lower jaw. Six weeks before the visit, she had gradually developed a herpes zoster eruption on the left side of her face in the distribution of the V2 and V3 branches of the trigeminal nerve. The patient was presented to the dermatology department and was treated with multiples oral medications, including antiviral drugs (valaciclovir hydrochloride tablets), anticonvulsants (pregabalin), and neurotrophic drugs (mecobalamin tablets). The skin vesicles gradually subsided after 2 weeks, but facial pain persisted. The physician suggested increasing the dosage of pregabalin to 225 mg two times per day and prescribed antidepressant agents (flupentixol and melitracen tablets 10.5 mg two times per day) for alleviating facial pain. Unfortunately, her pain was refractory to medical therapy, and her nocturnal sleep did not improve her during medication. The pain was assessed as 9/10 on the visual analog scale (VAS), and it could be triggered by usual touching stimulation. The Self-Rating Anxiety Scale (SAS) and the Self-Rating Depression Scale (SDS) were used to evaluate the anxiety and depression levels, respectively. The higher the score, the more severe the states of anxiety and depression. Both the SAS and SDS have been extensively used to assess pain-related anxiety and depression, demonstrating good validity and reliability (22, 23). The 12-item Short-Form Health Survey (SF-12) questionnaire was used to evaluate the quality of life (QoL), which was described from the aspects of Physical Component Summary (PCS) and Mental Component Summary (MCS). Higher scores indicate better health status. The result showed that the patient experienced severe facial pain, as well as mild anxiety and depression, which significantly affected the QoL. During physical examination upon admission, hyperalgesia and skin pigmentation on her left V2 and V3 branches of the trigeminal nerve were confirmed. Magnetic resonance imaging of the skull base showed normal results without space-occupying lesions. Additionally, the patient had a history of hypertension but no other neurological or chronic pain conditions. Therefore, the patient was diagnosed with subacute herpetic trigeminal neuralgia involving theV2 and V3 branches, and she had to undergo temporary GGS treatment utilizing a quadripolar-tined SNM lead for pain relief.

**Figure 2:**
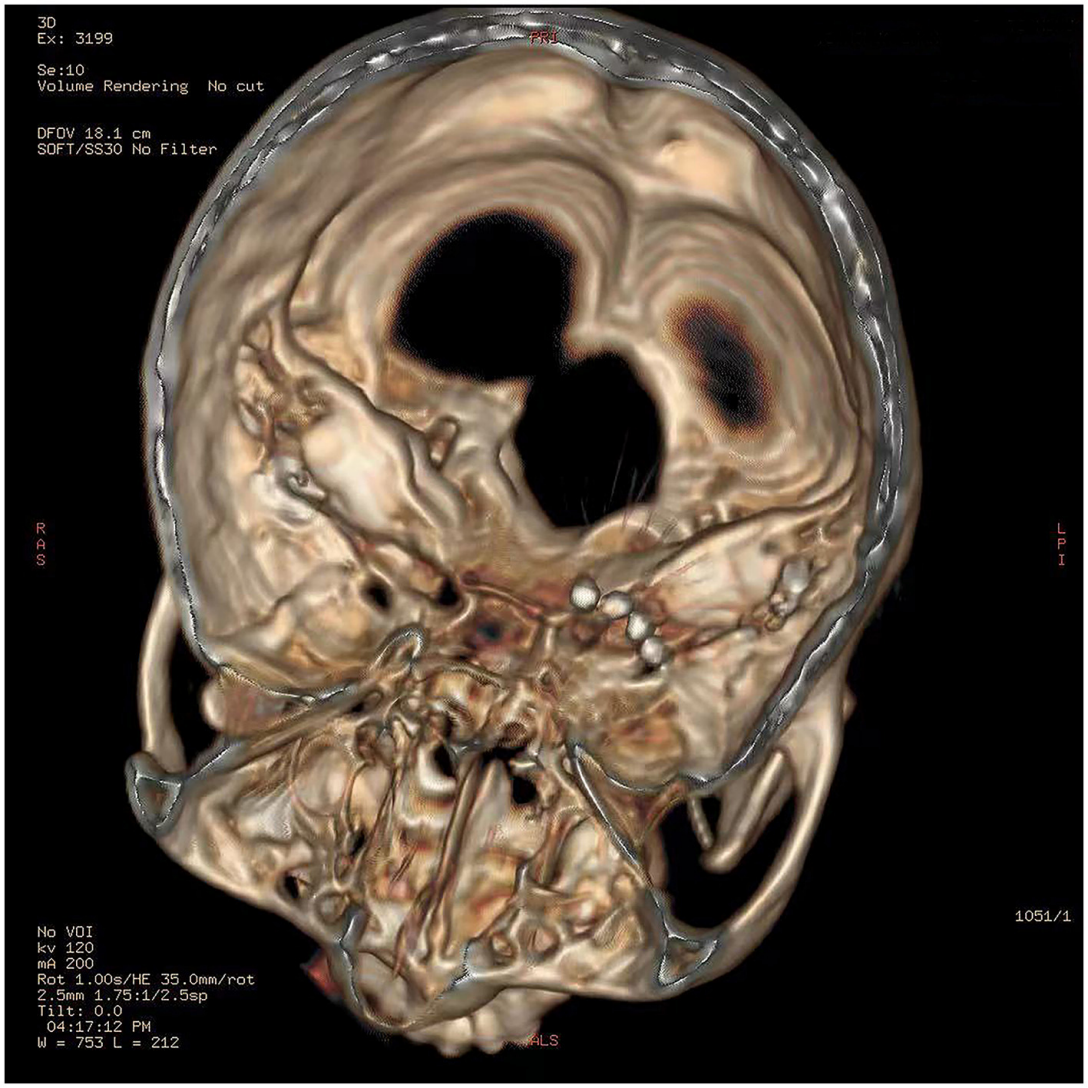
The electrode remained in the correct position under CT reconstruction.

The procedures of the surgery included computed tomography (CT)-guided percutaneous foramen ovale (FO) puncture and temporary electrode implantation. Before the operation, the patient was resting in a supine position and her head was kept in a reverse occipitomental position. Vital signs such as heart rate, blood pressure, and oxygen saturation were monitored in the operating room. A 3D-CT scan of the head was performed to determine the FO and puncture site. After the puncture route was scheduled optimally, the patient underwent sterile preparation, and local anesthesia was given to her. Then, an 18-G puncture needle was advanced into the FO following the Hartel anterior approach. The needle tip entered the FO under fluoroscopy, through which the quadripolar self-blocking tined lead (Model 3889 lead) was inserted. After confirming that the electrode remained in the correct position under CT reconstruction (Figure 2), the needle was withdrawn and the tines were released and attached to the soft tissue inferior to the FO and did not enter the foramen. Once the tined lead was positioned, the next step was to connect to an external test stimulator (Model 3625 test stimulator, Medtronic) for testing. The implanted electrode was programmed in tonic mode, with a pulse width of 210 μs, a frequency of 15 Hz, an amplitude of 1.4 V, and the contact polarity set to 1+ and 3–. Paresthesia was elicited in both the V2 and V3 branches by stimulation, covering the primary pain regions including the nose, zygomatic zone, lower jaw, and lower lip. Then, the patient was returned to the ward with the lead and wire fixed properly and received GGS for 14 days.

**Figure 3:**
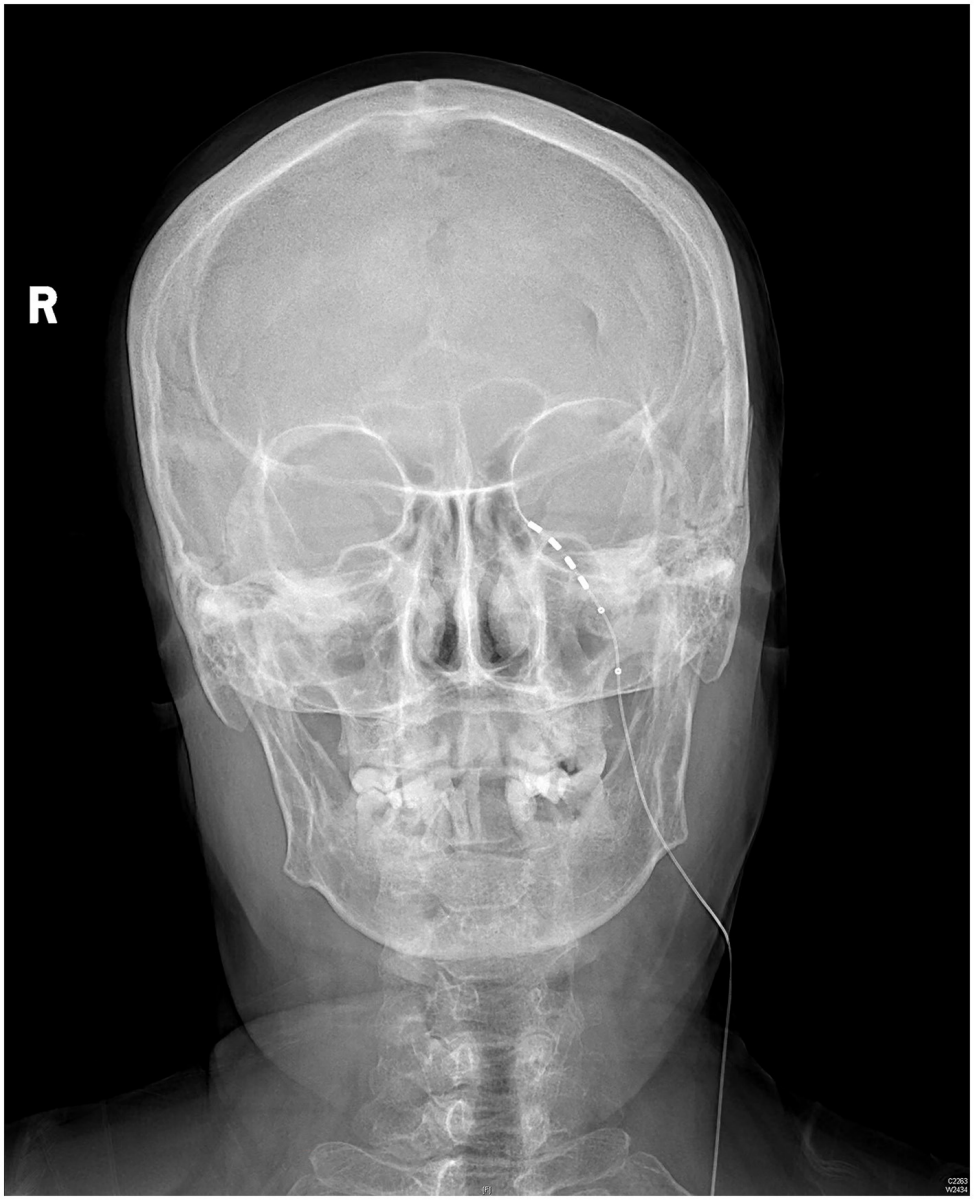
A skull-base x-ray of the anteroposterior view.

**Figure 4:**
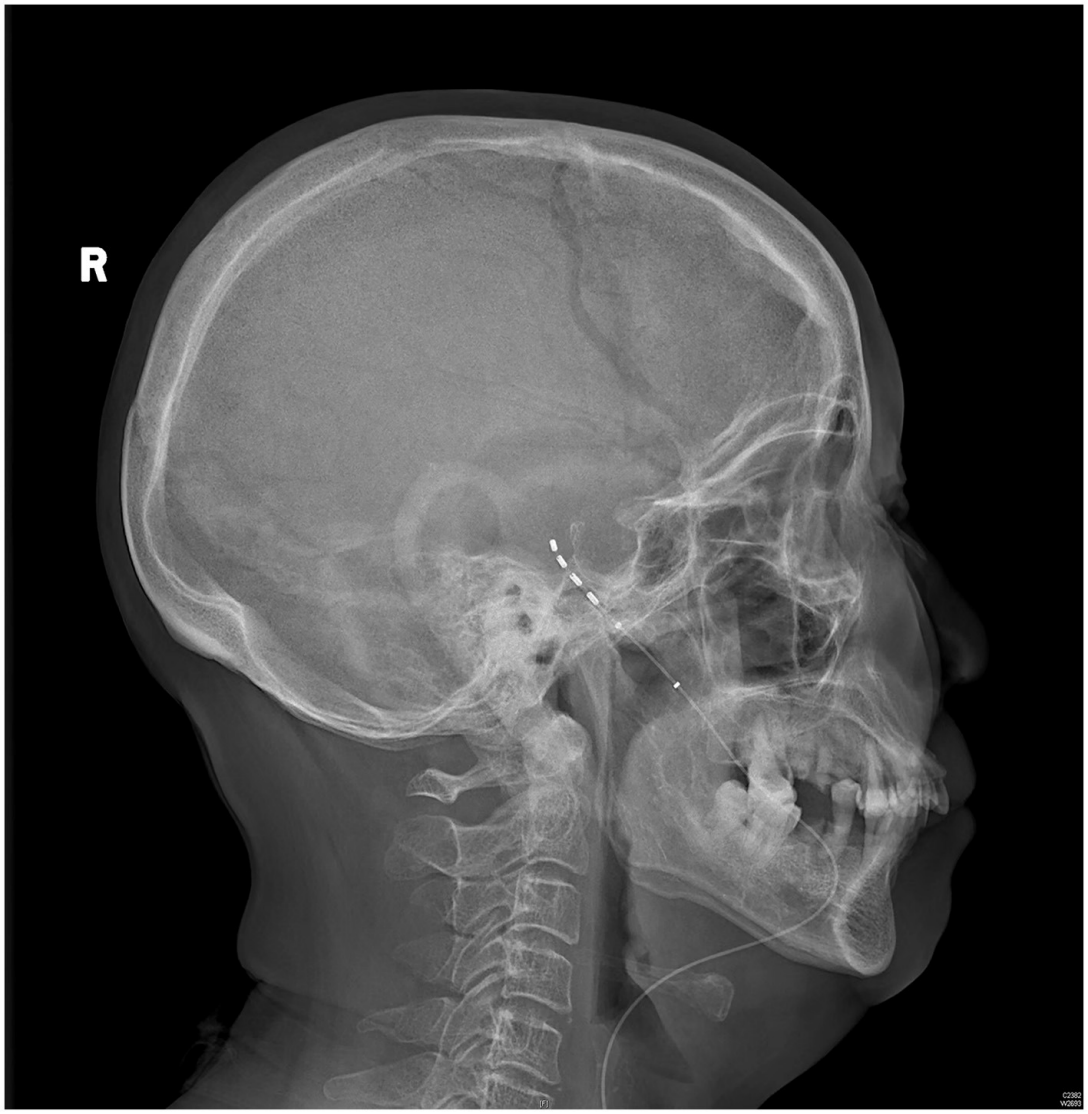
A skull-base x-ray of the lateral view.

The patient rated her facial pain on the VAS as 3/10 with paresthesia sensation on the left side of her face the day after implantation. The pain reduced to 1/10 on the VAS 3 days after implantation. After the 14-day stimulation, the patient underwent a skull-base x-ray scan (Figures 3, 4), which revealed that the quadripolar electrode had bent over the Gasserian ganglion without dislocation. Furthermore, after the patient rated that satisfactory pain relief was achieved with pregabalin, the dosage was reduced to 75 mg at night and the electrode was pulled out. SDS, SAS, and SF-12 were evaluated again before discharging the patient. The results indicated a significant improvement in pain as compared to before surgery. VAS, oral medication, and complications were followed up through telephone interviews at 1, 3, 6, and 12 months by the pain physician (Table 1). At the 12-month follow-up after surgery, the patient's VAS score was 4/10, and the patient was taking 75 mg of pregabalin orally per night. Although a small increase was noted in the VAS score at the 12-month follow-up after surgery compared to the VAS score at the time of discharge, the patient's trigeminal pain decreased by over 50% compared to the VAS score at the time of admission, which indicated that stable and satisfactory pain relief was achieved through temporary GGS.

**Table 1:** VAS, SDS, SAS, SF-12, oral medication, and complications in follow-up.

**Variable**	**Pre-surgery**	**1 day**	**3 days**	**7 days**	**14 days**	**1 month**	**3 months**	**6 months**	**12 months**
VAS	9	3	1	1	1	1	1	4	4
SDS	62	–	–	–	40	–	–		
SAS	58	–	–	–	35	–	–		
PCS of SF-12	33.9	–	–	–	47.0	–	–		
MCS of SF-12	27.4	–	–	–	41.9	–	–		
Pregabalin dose/per day	450 mg	450 mg	300 mg	150 mg	75 mg	75 mg	75 mg	75 mg	75 mg
Flupentixol and melitracen dose/per day	21 mg	21 mg	21 mg	10.5 mg	10.5 mg	5.25 mg	0 mg	0 mg	0 mg
Complications	–	Paresthesia	Paresthesia	Paresthesia	Paresthesia	–	–	–	–

VAS, visual analog scale; SDS, Self-Rating Depression Scale; SAS, Self-Rating Anxiety Scale; SF-12, 12-item Short-Form Health Survey; PCS, Physical Component Score; MCS, Mental Component Score.

## 3 Discussion

Herpetic neuralgia can be defined in three stages: AHN, SHN, and PHN (9). AHN refers to herpes zoster-related pain that is experienced within 1-month of the rash onset. SHN refers to herpes zoster-related pain that persists over 1 month but not more than 3 months. If neuropathic pain continues to persist for more than 3 months after the onset of herpes zoster rash, then it should be considered PHN. If the zoster-related pain is not under control during the stages of AHN or SHN, the patient might experience prolonged pain, increasing the risk of developing PHN even after the rash has healed. Furthermore, herpetic trigeminal neuralgia is also a challenging and refractory form of trigeminal neuropathy to treat. Although first-line therapy involves medication treatment, it has adverse effects such as nausea, vomiting, and dizziness, which can be intolerable for elderly patients, especially for those with comorbidities such as hypertension or diabetes mellitus (24, 25). Interventional treatment is considered when patients fail to respond to conservative medication therapy. However, it is difficult to benefit from traditional treatments for trigeminal neuralgia, such as microvascular decompression, trigeminal ganglion radiofrequency thermocoagulation, and gamma knife surgery, when treating trigeminal neuropathy. Previous studies have reported undesirable outcomes where neuro-destructive interventions had little beneficial effect and exacerbated pain symptoms in 73% of patients (26). Furthermore, neuro-destructive surgery was also not recommended for patients with trigeminal neuropathy in other studies (19, 20). The preferred and alternative forms of treatment for herpetic trigeminal neuralgia include neuromodulation techniques, such as pulsed radiofrequency, peripheral trigeminal nerve stimulation, or GGS. Pulsed radiofrequency (PRF) has been used for the treatment of herpetic trigeminal neuralgia depending on its nerve modulation function. Some studies illustrated that PRF was an effective and safe method for both acute/subacute herpetic trigeminal neuralgia and postherpetic trigeminal neuralgia, with a total efficiency rate ranging from 68.9 to 86.7% (27, 28). However, there was some controversy about the PRF treatment for herpetic neuralgia. A systematic review indicated that PRF was not initially recommended considering the aspects of invasiveness, price, and safety (29). Despite the lack of high-level evidence, PNS and GGS are the probable and preferred treatments for herpetic trigeminal neuralgia (29). The possible mechanism may be related to the normalization of sensation and gate control theory. Viral infection and reactivation remaining in the Gasserian ganglion can impair the trigeminal nerve. The Gasserian ganglion contains sensory afferent neuron bodies, similar to those found in the dorsal root ganglia, that are essential for transmitting signals from the peripheral trigeminal nerves to the central nervous system (30). Lazorthes et al. observed changes in the sensory function of the trigeminal nerve through GGS and revealed that GGS played the function of sensory normalization and pain improvement, which might be the potential mechanism (31). Gate control theory indicates that activating large-caliber non-nociceptive A-fiber afferent nerves could regulate the nociceptive transmission pathway, and proper stimulation of these A-fiber nerves could inhibit or reduce pain signal transmission (32). Another explanation might be that the stimulation activates descending inhibitory pathways and increases the release of inhibitory neurotransmitters (e.g., GABA), thereby suppressing pain signals (33).

However, we found that many studies have mainly focused on permanent electrode implantation for trigeminal neuropathy, possibly due to stroke- or surgery-induced trigeminal nerve lesions, which account for the majority (6). For patients in the early stage of herpetic trigeminal neuralgia, few studies have reported the efficacy of temporary GGS (6). Prompt and effective measures of pain control and hyperalgesia prevention should be the priority. We reported a patient with SHN involving the V2 and V3 branches of the trigeminal nerve who underwent temporary GGS for 14 days utilizing an SNM electrode. The patient was discharged with a VAS score of 1/10; improved anxiety and depression; enhanced QoL; and reduced oral medications. The 12-month follow-up also showed stable pain relief (VAS 4/10) after the electrode was pulled out. The studies by Liu et al. and Huang et al. revealed that the VAS score was significantly improved in patients with AHN or SHN during the 6–12 month follow-up period among those undergoing early PNS and SCS treatment (4, 9); this finding was consistent with the result of our study. The authors also stressed the importance of early PNS and SCS treatment for herpetic neuralgia.

Long-term GGS has been used to treat refractory herpetic trigeminal neuralgia, and permanent electrode implantation seems to be necessary to alleviate prolonged facial pain experienced by patients if the trial stimulation is satisfactory. Nevertheless, the issue of whether permanent electrode implantation is indispensable is worth considering. Lise and Zhao's studies reported that, in spite of the promising results of initial permanent GGS, less than half of the patients experienced partial pain relief in the long run (24 months) (14, 20). Moreover, electrode dislocation (10–30%) can lead to inadequate pain coverage, and long-term dysesthesia or physical discomfort (41%) might be intolerable for patients (19, 20). In addition, the cost of an impulse generator for permanent implantation is approximately equivalent to USD $25,000, which can pose a significant financial burden on elderly patients. Based on our case report, temporary GGS could be an alternative treatment. We speculate that the encouraging results of our case are attributable to two aspects: early stimulation and electrode choice. Neuromodulation is recommended for persistent pain lasting over 6 months and refractory to conservative therapies (34). However, if herpes zoster-related pain progresses to the PHN stage, particularly trigeminal PHN, it can be very difficult and frustrating for pain physicians to treat it. Previous studies have shown that the therapeutic effect of SCS is associated with the timing of pain onset. It is believed that the shorter the time from pain onset to stimulation, the better the effect. With regard to GGS, a technique with a mechanism similar to SCS, earlier interventional treatment seems to have a more positive effect on patients with AHN or SHN. The clinical observation of prolonged pain relief in our case could be explicated by peripheral and central sensitization reversal in the early stages of neuropathic pain. It has been recognized that nerve injury-induced inflammatory factors sensitize nociceptors and amplify the afferent pain signals. Meanwhile, the persistent stimulus can abnormally increase the sensitivity and excitability of nociceptive neurons in both the spinal cord and the brain within the central nervous system (35). Performing short-term GGS could normalize and improve pain sensation and even have an impact on cerebral pain modulation, thus inhibiting the development of herpetic trigeminal neuralgia. Moreover, the effect of GGS depends on the degree of damage to the trigeminal nerve (36). The less the trigeminal impairment, the better the pain improvement, which indicates the importance of early GGS. In terms of the electrode choice for temporary GGS, electrode migration was associated with inadequate stimulation coverage and reduced therapeutic effect. Electrode dislocation was related to the specific anatomy of the electrode path toward the FO. The tripolar tined lead (Model 09053, Medtronic Neurological) is a good option, but it is not available in China. We must utilize the existing electrode to solve the problem of dislocation. Owing to the anti-dislocation effect of tines in the SNM electrode, the patient revealed that the current coverage was consistent with the primary facial pain region without other complications except for paresthesia. Paresthesia also disappeared when the electrode was pulled out. It is important to note that tines on the SNM lead can help anchor the lead, but they can also prevent the lead from being fully removed, which was our one of our concerns when this lead was first applied. However, our previous cohort study on sacral nerve stimulation using SNM lead in patients with pudendal neuralgia did not find any complications related to lead breakage during removal (37). In addition, it is possible to avoid electrode breakage by employing appropriate surgical techniques (38). Therefore, the quadripolar tined lead designed for SNM might be a safe and suitable choice for herpetic neuralgia.

Our case report had several limitations that should be addressed in future studies. First, this was only a case report with a 12-month follow-up period. The results should be considered preliminary clinical findings. Large-scale, prospective, and randomized studies with further follow-up are needed to validate our assumption. Second, telephone follow-ups did not allow for quantitative evaluation of the complications or side effects, relying solely on the patient's subjective reports. Third, quantitative sensory testing, an electrophysiological method for objectively evaluating the sensory nerve function, should be included in subsequent case series.

## 4 Conclusion

Temporary GGS might be a potentially effective treatment for subacute herpetic trigeminal neuralgia. In combination with reduced dosages of pregabalin, it reduces hyperalgesia and refractory pain. The application of an SNM electrode might reduce the risk of dislocation. Further studies should validate our findings through large-scale randomized controlled trials.

## Data availability statement

The raw data supporting the conclusions of this article will be made available by the authors, without undue reservation.

## Ethics statement

The studies involving humans were approved by Ethics Committee of Beijing Tsinghua Changgung Hospital (23208-4-02). The studies were conducted in accordance with the local legislation and institutional requirements. The participants provided their written informed consent to participate in this study. Written informed consent was obtained from the individual(s) for the publication of any potentially identifiable images or data included in this article.

## Author contributions

JN: Conceptualization, Investigation, Methodology, Writing – original draft. CW: Investigation, Methodology, Writing – original draft. XW: Project administration, Software, Writing – original draft. GL: Investigation, Methodology, Project administration, Supervision, Writing – original draft, Writing – review & editing.
